# Supplementary material for: Quantitative models reveal the organization of diverse cognitive functions in the brain
Source: Nat Commun. 2020 Mar 2;11:1142. doi: 10.1038/s41467-020-14913-w (PMC7052192; doi:10.1038/s41467-020-14913-w)
Supplement: Supplementary file 1 — Supplementary Information [file 41467_2020_14913_MOESM1_ESM.pdf]

Supplementary Information

Quantitative models reveal the organization of diverse cognitive functions in the brain

Nakai and Nishimoto

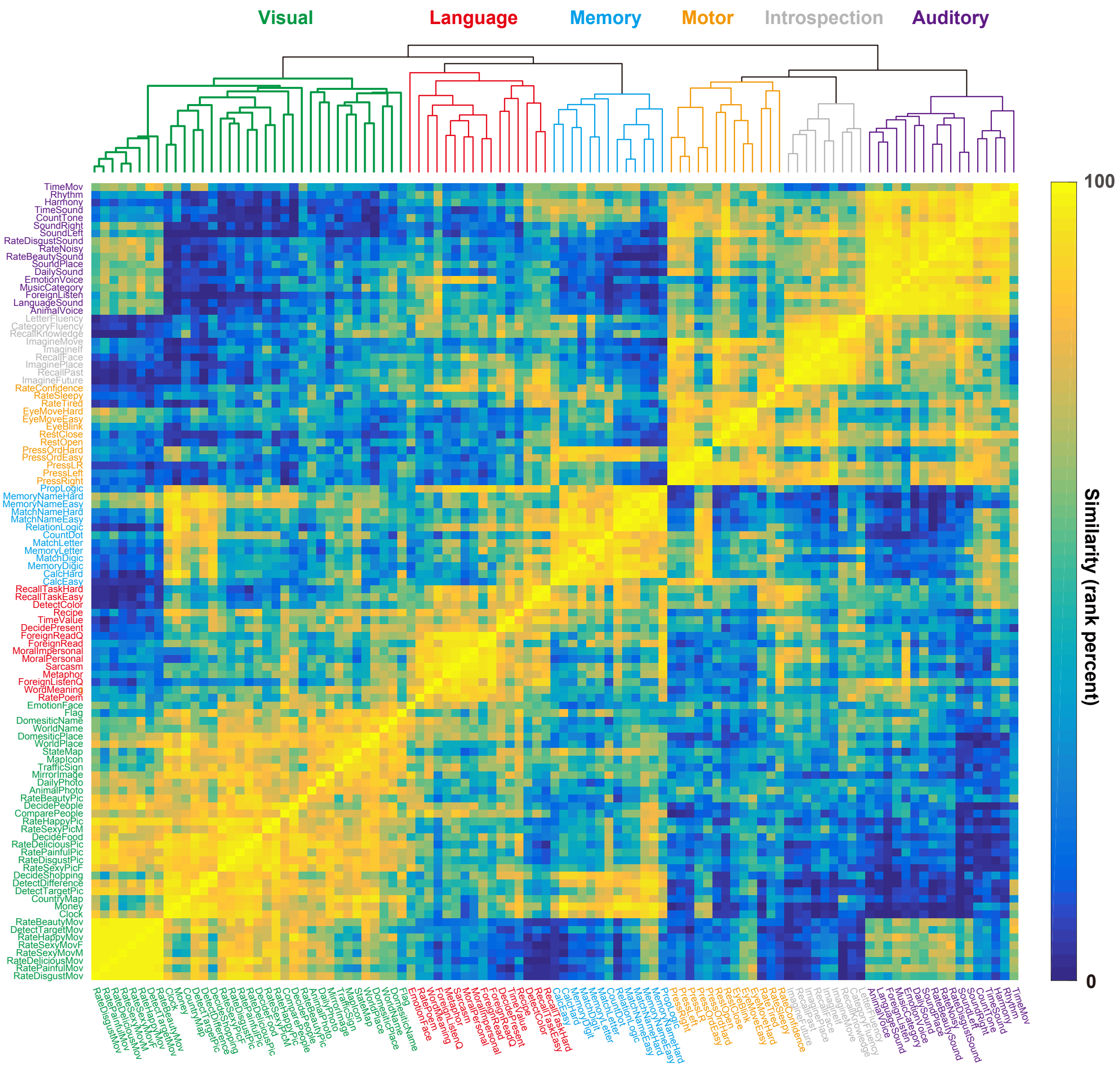

Supplementary Figure 1. Hierarchical cluster analysis using brain activity. a, Representational similarity matrix of the 103 tasks, reordered according to the hierarchical cluster analysis (HCA) using brain activity of the whole cerebral cortex (concatenated across subjects). The dendrogram shown in the top panel represents the results of the HCA. The six largest clusters were named after the included task types.

Supplementary Note 1. Hierarchical cluster analysis using brain activity

To investigate task clusters in a model-independent way, we conducted hierarchical clustering analysis (HCA) using brain activity of the whole cerebral cortex, concatenated across six subjects. Brain activity during the task period with three time delays was averaged for each task. The representational similarity matrix (RSM) and dendrogram were obtained following the same procedure as the HCA using a task-type weight matrix. Each cluster was labeled according to the cognitive tasks included. The resultant RSM and dendrogram (Supplementary Figure 1) were very similar to those obtained using the task-type weight matrix (Fig. 3).

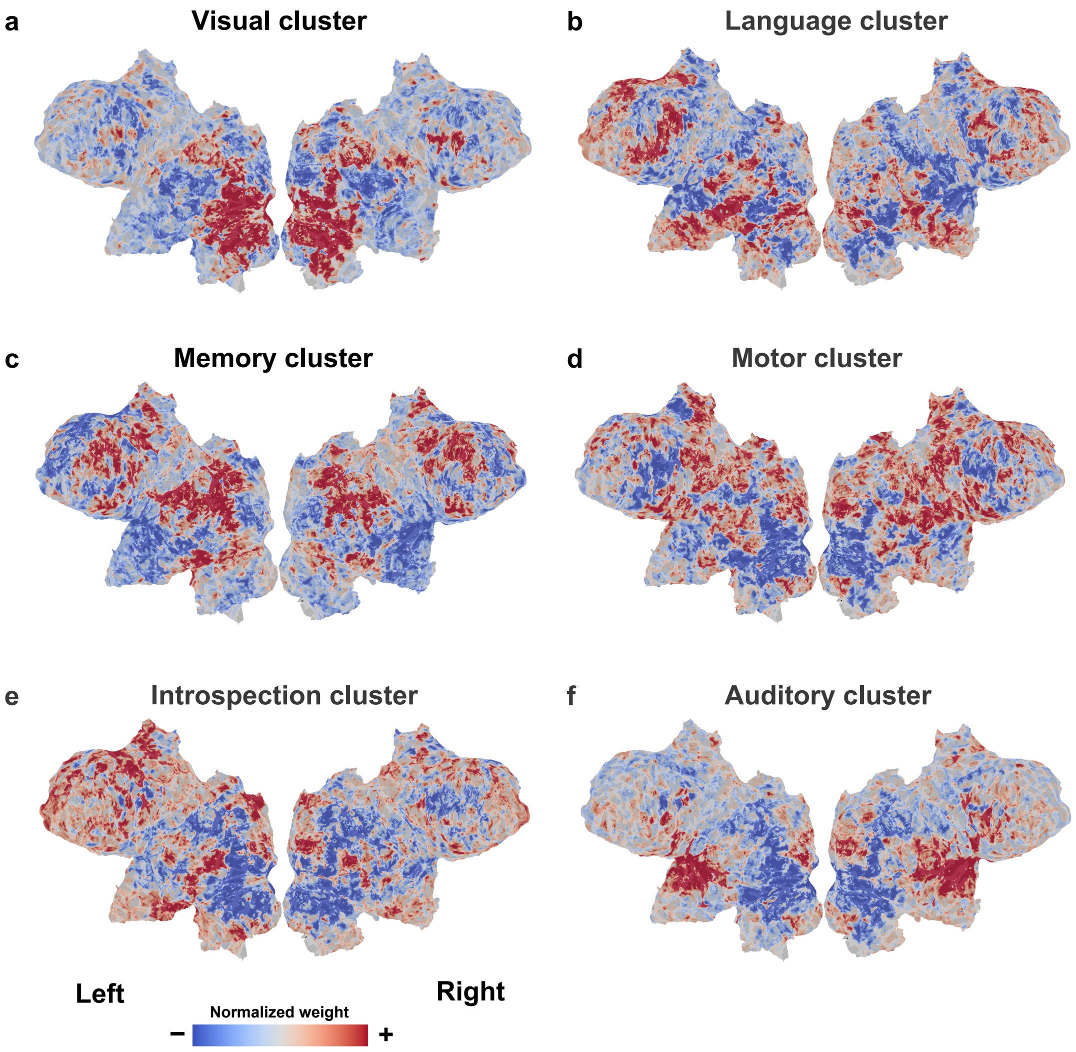

Supplementary Figure 2. Cortical maps of the task clusters. Normalized cortical maps of the weight matrices using the task-type model, showing the visual (**a**), memory (**b**), language (**c**), motor (**d**), introspection (**e**), and auditory (**f**) clusters.

Supplementary Note 2. Cortical representation of the task clusters

To assess the brain regions associated with each task cluster, we examined the weight matrix for only those tasks that were within each of the six largest task clusters (Supplementary Figure 2). The weight values of the target clusters were then averaged across the tasks and normalized across voxels. This indicated the relative contribution of each cortical voxel to the target task cluster. Accordingly, the task cluster related to visual processing showed large weights in the occipital regions, the cluster related to auditory processing showed large weights in the superior temporal regions, and the one related to memory processing showed large weights in the frontal and parietal regions. The language cluster showed large weights in the left frontal and inferior

temporal regions, the motor cluster showed large weights in the pericentral regions, and the task cluster that was related to introspective processes showed large weights in the medial frontal and cingulate regions. These cluster weight maps were further used to evaluate the cognitive factors related to each task cluster.

Supplementary Table 1. Top cognitive factors related to subclusters

	Top cognitive factors in the Neurosynth database
Time-perception subcluster	"auditory," "sound," "pitch," "acoustic," "speech," "music," "sensory," "working memory," "timing," "monitoring"
Knowledge-recalling subcluster	"phonological," "motor," "production," "overt", "language," "word," "lexical," "verb," "articulatory," "preparation"

Selected cognitive factors from top 50 cognitive factors in the Neurosynth database for two subclusters, based on the correlation coefficients between each subcluster weight map and the 715 registered reverse-inference maps.

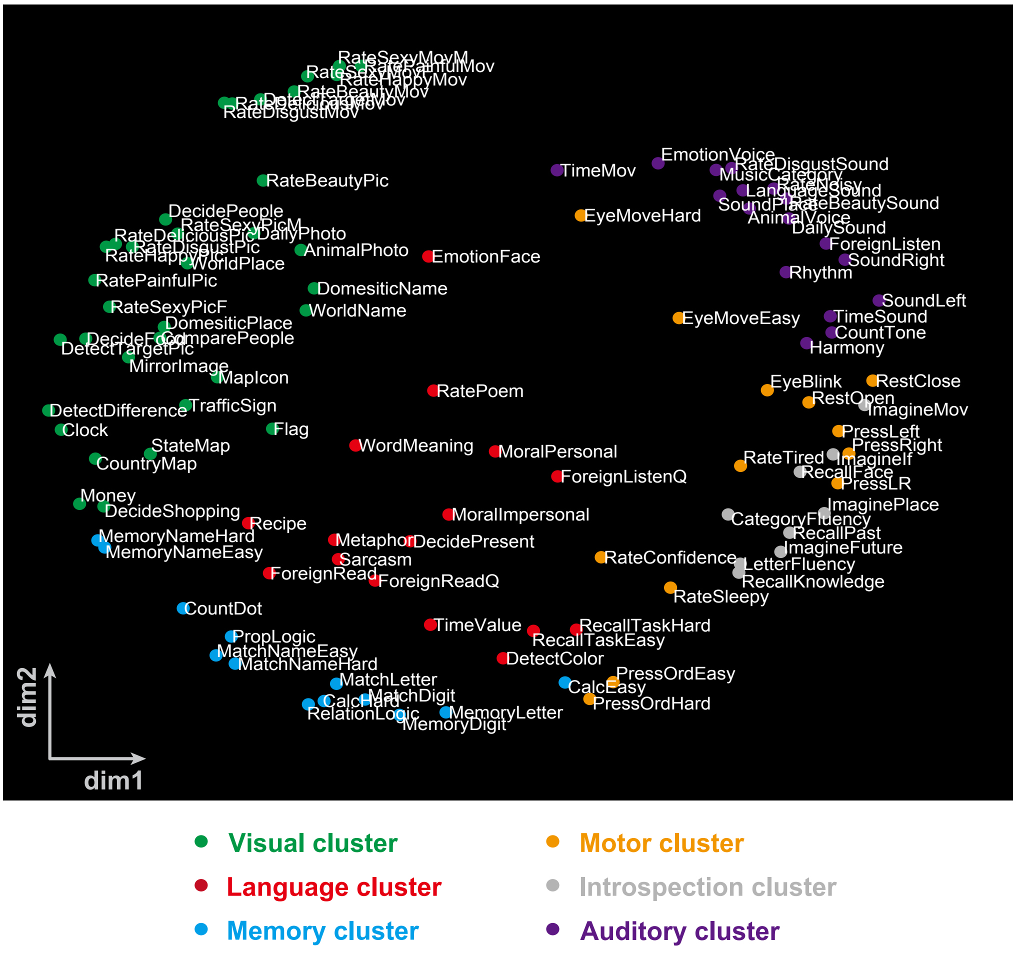

Supplementary Figure 3. Visualization of cognitive space using multi-dimensional scaling. A total of 103 tasks were mapped onto the two-dimensional cognitive space based on the 1st and 2nd dimensions obtained using a non-metric multi-dimensional scaling. Colors indicate the six largest clusters depicted in Supplementary Figure 1. All task names are labeled in white.

Supplementary Note 3. Visualization of over 100 tasks using multi-dimensional scaling

To further investigate the representational relationship among the 103 tasks in a model-independent way, we conducted a non-metric multi-dimensional scaling analysis and visualized 103 tasks on the two-dimensional space based on the 1st and 2nd dimensions (Supplementary Figure 3). We found that tasks in the same cluster (obtained in the HCA in Supplementary Figure 1) were located closely and that the task organization was similar to that obtained using principal component analysis (PCA) (Fig. 4).

Supplementary Table 2. Top tasks related to each principal component

	Top 10 tasks
PC1 (auditory)	“SoundLeft,” “ForeignListen,” “SoundRight,” “CountTone,” “RestClose,” “Harmony,” “RateBeautySound,” “ImagineMove,” “Rhythm,” “RateNoisy”
PC2 (audiovisual)	“RateSexyMovF,” “RatePainfulMov,” “RateHappyMov,” “RateDisgustMov,” “RateSexyMovM,” “RateDeliciousMov,” “RateBeautyMov,” “DetectTargetMov,” “RateBeautySound,” “RateNoisy”
PC3 (language)	“MoralPersonal,” “Sarcasm,” “MoralImpersonal,” “ForeignListenQ,” “WordMeaning,” “ForeignReadQ,” “Metaphor,” “DecidePresent,” “RatePoem,” “DomesticName”
PC4 (introspection)	“ImagineMove”, “ImaginePlace,” “ImagineIf,” “RecallPast,” “RestOpen,” “PressLeft,” “PressRight,” “EyeBlink,” “PressLR,” “RecallFace”

Top 10 tasks with the largest PCA loadings for PC1 to PC4.

Supplementary Table 3. Top cognitive factors related to each principal component

	Top cognitive factors in the Neurosynth database
PC1 (auditory)	“auditory,” “sound,” “listening,” “speech,” “acoustic,” “pitch,” “audiovisual,” “speech perception,” “music,” “vocal”
PC2 (audiovisual)	“auditory, “pitch,” “sound,” “acoustic,” “listening,” “audiovisual,” “music,” “hearing,” “multisensory,” “tone”
PC3 (language)	“comprehension,” “semantic,” “sentence,” “language,” “linguistic,” “syntactic,” “word,” “reading,” “lexical,” “spoken”
PC4 (introspection)	“default mode,” “default network,” “motor,” “somatosensory,” “sensorimotor,” “self,” “hand,” “finger,” “autobiographical,” “movement”

Top 10 cognitive factors (excluding similar terms) in the Neurosynth database for PC1 to PC4, based on the correlation coefficients between each PC score map and the 715 registered reverse-inference maps.

Supplementary Note 4. Interpretation of cognitive factors related to principal components

To interpret the plausible cognitive factors related to the PCs, we used Neurosynth (http://neurosynth.org; accessed 26 January 2018) as a metadata reference of the past neuroimaging literature[1]. Each reverse-inference map in the Neurosynth database in MNI152 space was registered to the subjects’ reference echo-planar imaging (EPI) data using FreeSurfer[2,3]. For each PCA score map obtained with the task-type model, we calculated Pearson’s correlation coefficients between the PCA score map and the 715 registered reverse-

inference maps, resulting in a cognitive factor vector with 715 elements. Terms with higher correlation coefficient values were considered as contributing more to the target PC.

Several top PCs distinguished a class of sensorimotor components to the others, such as auditory (PC1; tasks with high loading, e.g., “SoundLeft,” “Harmony”) and audiovisual (PC2; “RatePainfulMov,” “RateHappyMov”), whereas the other PCs distinguished higher-order cognitive components, such as language (PC3; “Sarcasm,” “WordMeaning”), and introspection components (PC4; “ImagineMove,” “ImaginePlace”) (Supplementary Table 2).

The top 10 terms for each PC provided an interpretation of the relevant cognitive components, with PC1 (auditory) showing a high correlation with auditory-related terms (e.g. “auditory,” “sound”), PC2 (audiovisual) showing a high correlation with auditory- and vision-related terms (e.g. “audiovisual,” “multisensory”), and PC3 (language) showing a high correlation with language-related terms (“language,” “sentence”) (Supplementary Table 3). These results were largely consistent with the interpretations based on the high-loading task types.

For the other components, the interpretation based on Neurosynth provided a more detailed description. Although we labeled PC4 as the “introspection component” based on the high-loading task types, PC4 showed a high correlation with default mode-related terms in the Neurosynth database (“default mode,” “default network”). These results indicated that the metadata-based reverse-inference reliably captured the fundamental cognitive factors involved in the diverse cognitive tasks.

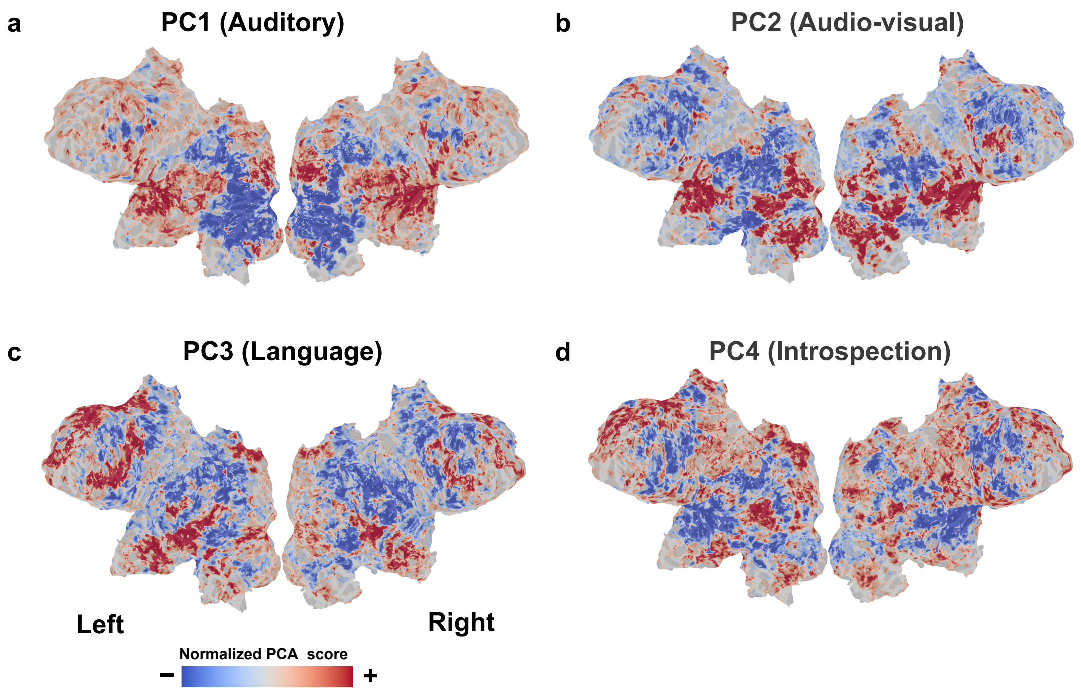

Supplementary Figure 4. PCA score maps. Normalized principal component analysis (PCA) scores of PC1–PC4 projected onto the flattened cortical sheets of subject ID01.

Supplementary Note 5. Cortical representation of principal components

To assess the cortical regions related to the top PCs, we projected normalized PCA scores onto the cortical maps (Supplementary Figure 4). PC1 (auditory component, according to Supplementary Table 2) had large weights in the superior temporal regions. PC2 (audiovisual component) had large weights in the superior temporal and occipital regions. PC3 (language component) had large weights in the frontal and inferior temporal regions. PC4 (introspection component) had large weights in the medial frontal and cingulate regions. These PCA score maps were further used to evaluate the cognitive factors related to each PC.

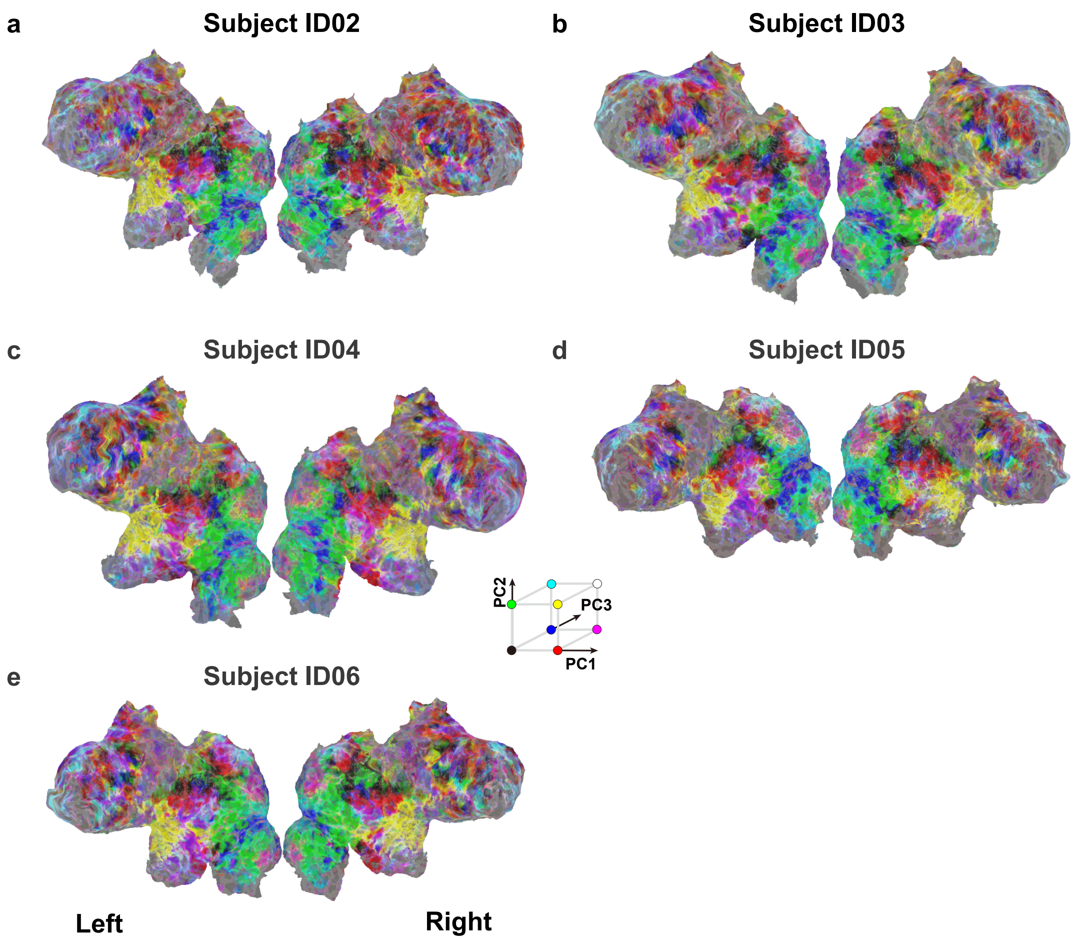

Supplementary Figure 5. Cortical mapping of the cognitive space. Cortical maps of the cognitive space are shown on the inflated and flattened cortical sheets of subjects ID02–ID06 visualized using scores of the top three principal components (PC1–PC3) in red, green, and blue, respectively.

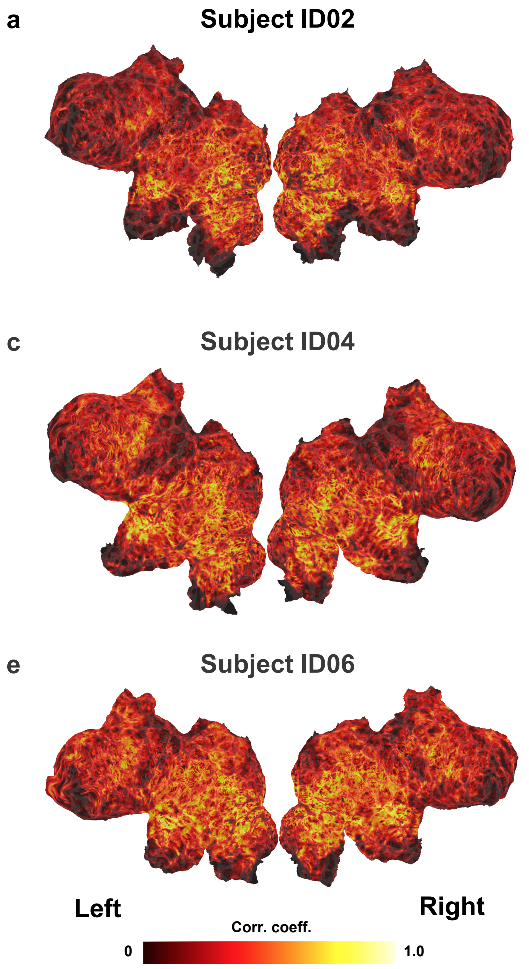

Supplementary Figure 6. Cortical map of prediction accuracy using the cognitive factor model under novel task conditions. The cortical map is shown on the flattened cortical sheets of subjects ID02–ID06 (mean prediction accuracy and percentage of significant voxels; ID02, 0.311 and 86.4%; ID03, 0.316 and 84.5%; ID04, 0.357 and 90.7%; ID05, 0.252 and 77.2%; ID06, 0.373 and 91.4%; $p < 0.05$, false discovery rate (FDR)-corrected; the minimum correlation coefficient for the significance criterion ranged from 0.0833 to 0.0872 for each individual subject).

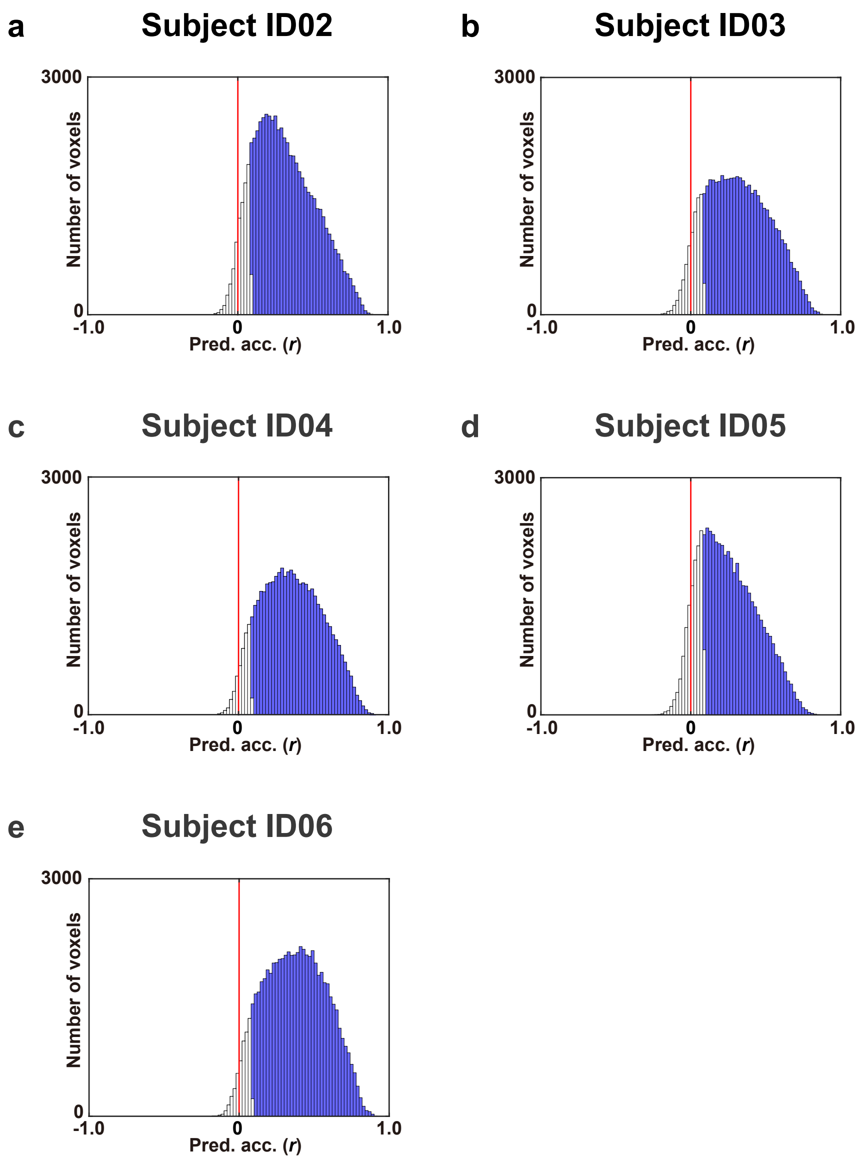

Supplementary Figure 7. Distribution of prediction accuracy using the cognitive factor model under novel task conditions. Histogram of prediction accuracies for all cortical voxels for subjects ID02-ID06. Filled bars indicate voxels that were predicted with significant accuracy.

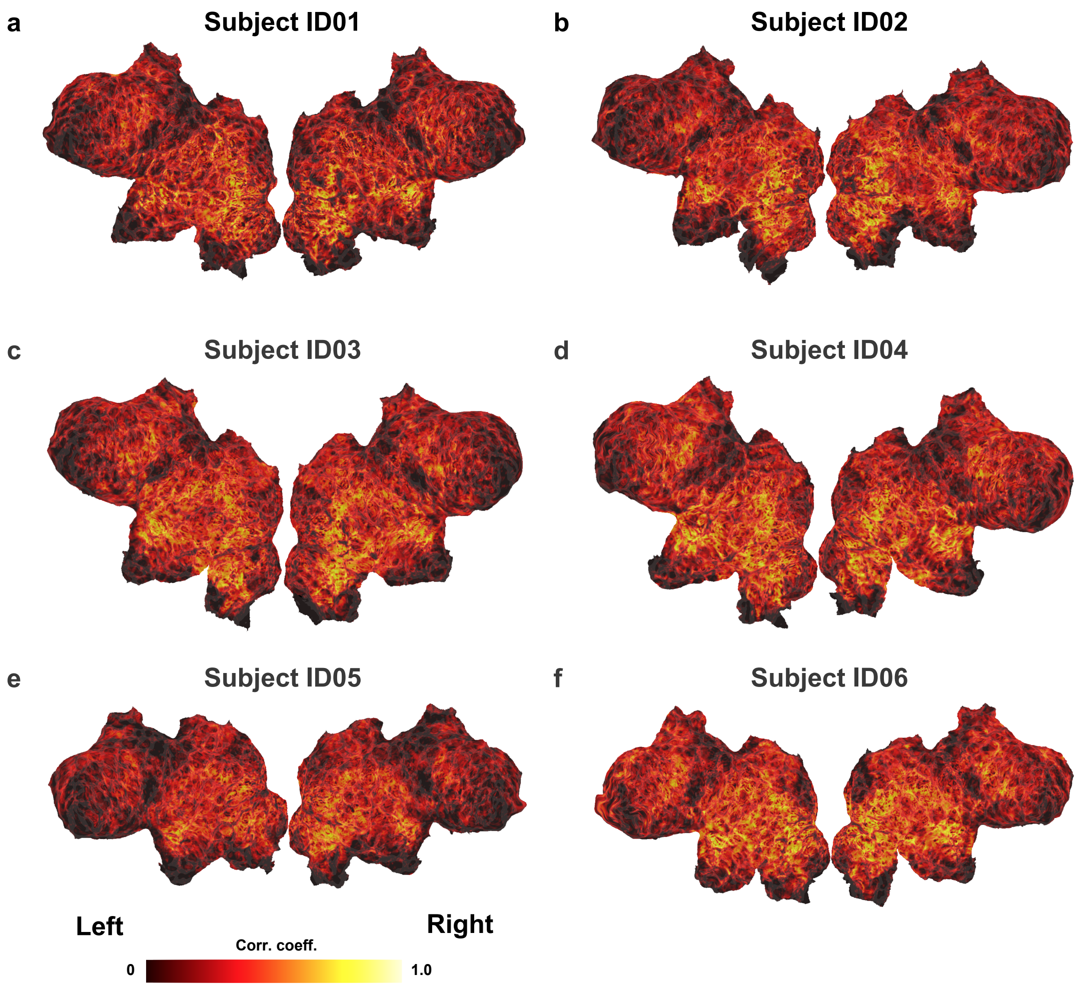

Supplementary Figure 8. Prediction accuracy of the cognitive factor model excluding sensorimotor features. The cortical map is shown on the flattened cortical sheets of subjects ID01–ID06 (mean prediction accuracy and percentage of significant voxels; ID01, 0.285, 82.3%; ID02, 0.273, 82.0%; ID03, 0.283, 81.7%; ID04, 0.315, 86.9%; ID05, 0.226, 73.9%; ID06, 0.327, 87.6%; $p < 0.05$, FDR-corrected; the minimum correlation coefficient for the significance criterion ranged from 0.0844 to 0.0881 for each individual subject).

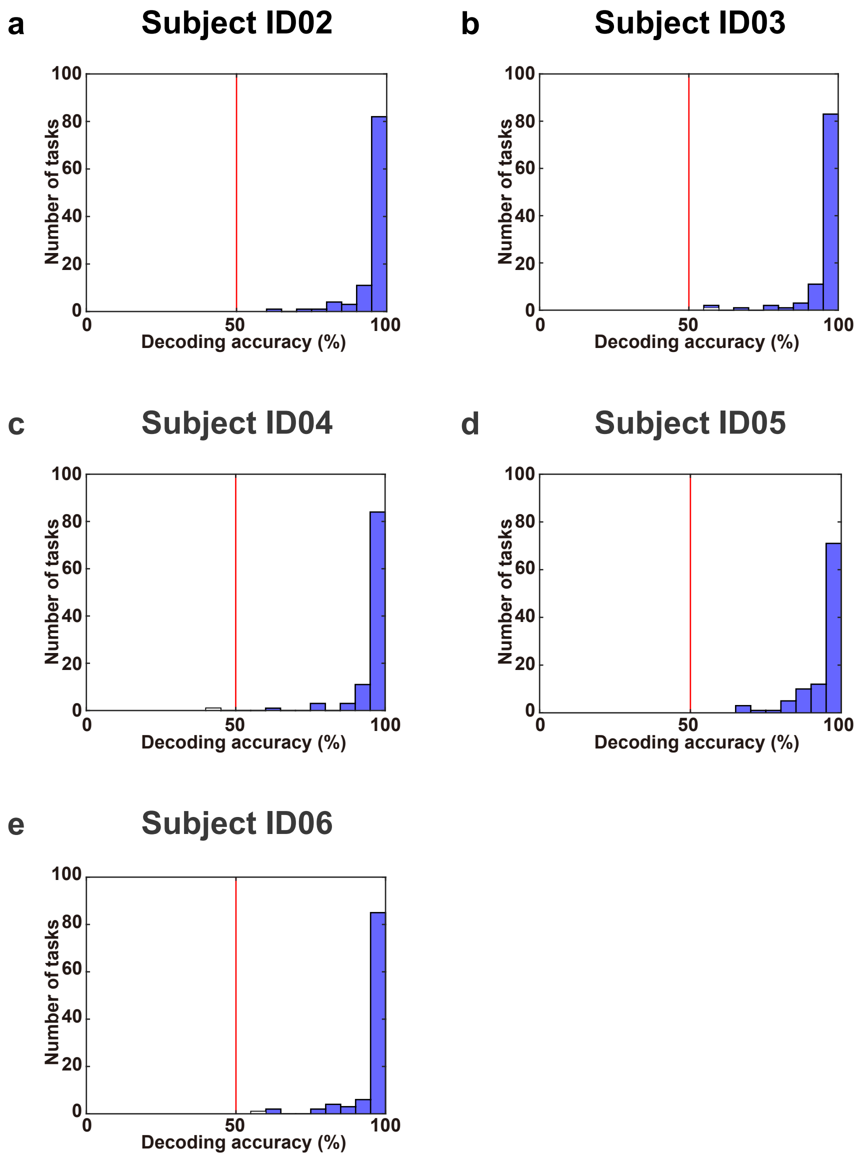

Supplementary Figure 9. Decoding of novel tasks. Histogram of decoding accuracies of over 100 tasks obtained using the cognitive factor model with novel tasks, for subjects ID02–ID06. The red line indicates the chance-level accuracy (50%). Filled bars indicate tasks that were decoded with significant accuracy (mean decoding accuracy and percentage of significant tasks; ID02, 98.3%, 100%; ID03, 96.0%, 100%; ID04, 96.4%, 100%; ID05, 94.6%, 100%; ID06, 95.9%, 99.0%; one-sided sign tests, $p < 0.05$, FDR-corrected).

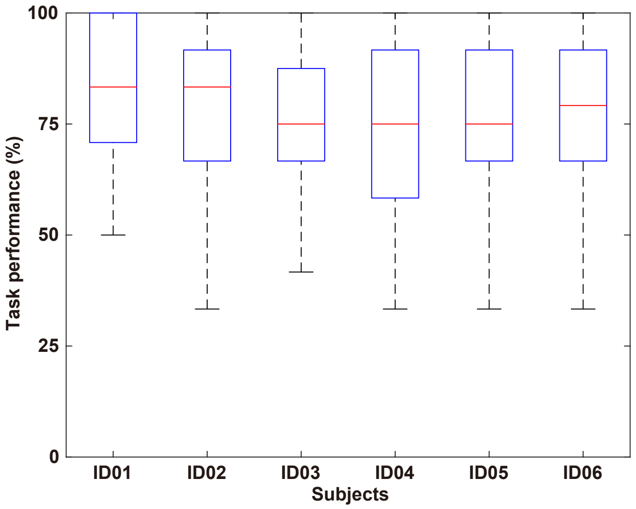

Supplementary Figure 10. Behavioral results. Box plots of accuracy for 48/103 tasks are shown for subjects ID01-ID06. Each box shows the median (red), the interquartile range (blue), and the maximum and minimum values.

Supplementary Note 6. Behavioral results

To show that the tasks used in the current study were sufficiently natural and easy to perform, we analyzed the behavioral performance for 48/103 tasks (Supplementary Figure 10). These 48 tasks were selected because only these tasks presented a single "yes or no" question. All subjects performed these tasks significantly better than at chance level (mean ± SD, 77.9 ± 2.8%; one-sided Wilcoxon signed-rank tests, $p < 0.05$, FDR-corrected), indicating that they understood the tasks without any preexperimental training or explanation. We also confirmed that the subjects did not have any difficulty in understanding the task settings via self-reports after the experiment.

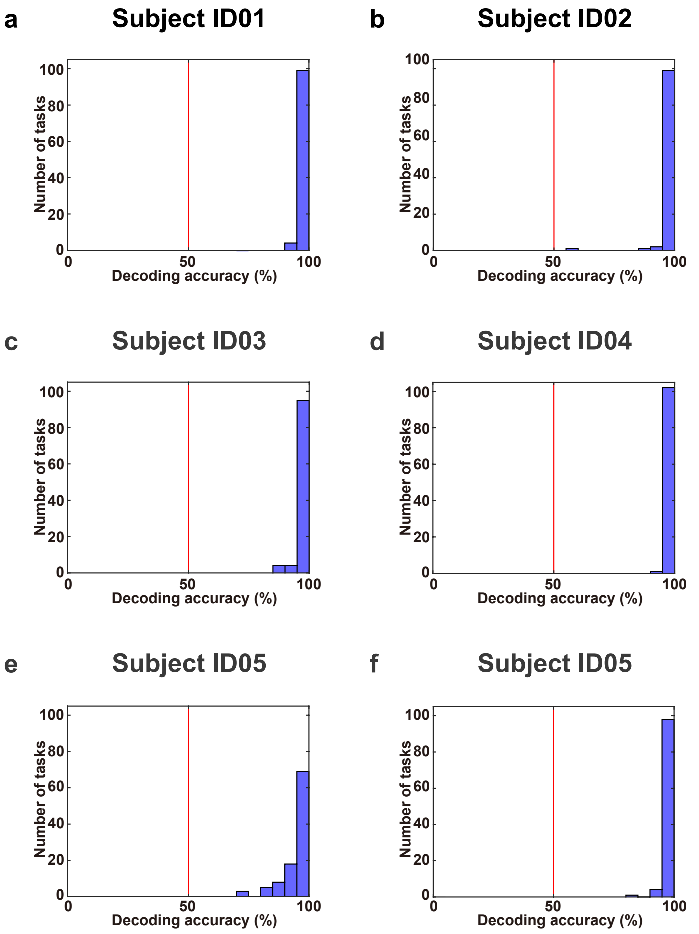

Supplementary Figure 11. Decoding directly from brain activity. Histogram of decoding accuracies of over 100 tasks obtained using a linear support vector machine for subjects ID01–ID06. The red line indicates the chance-level accuracy (50%). Filled bars indicate tasks that were decoded with significant accuracy (mean decoding accuracy and percentage of significant tasks; ID01, 99.0%, 100%; ID02, 98.1%, 100%; ID03, 98.0%, 100%; ID04, 99.1%, 100%; ID05, 94.5%, 100%; ID06, 98.6%, 100%; one-sided sign tests, $p < 0.05$, FDR-corrected).

Supplementary Note 7. Decoding tasks directly from brain activity

To further test whether we can decode over 100 tasks in a less model-dependent way, we also applied a linear support vector machine (SVM) analysis directly to the task-evoked brain activity. We used LIBSVM for the linear SVM decoding of 103-way multi-class classification[4]. The SVM decoder was constructed based on the response matrix and the task-label vector with an index of 1–103. This analysis exhibited high decoding accuracy for all subjects (mean ± SD, 97.9 ± 1.7%; all tasks were significant; one-sided sign tests, $p < 0.05$, FDR-corrected; Supplementary Figure 11).

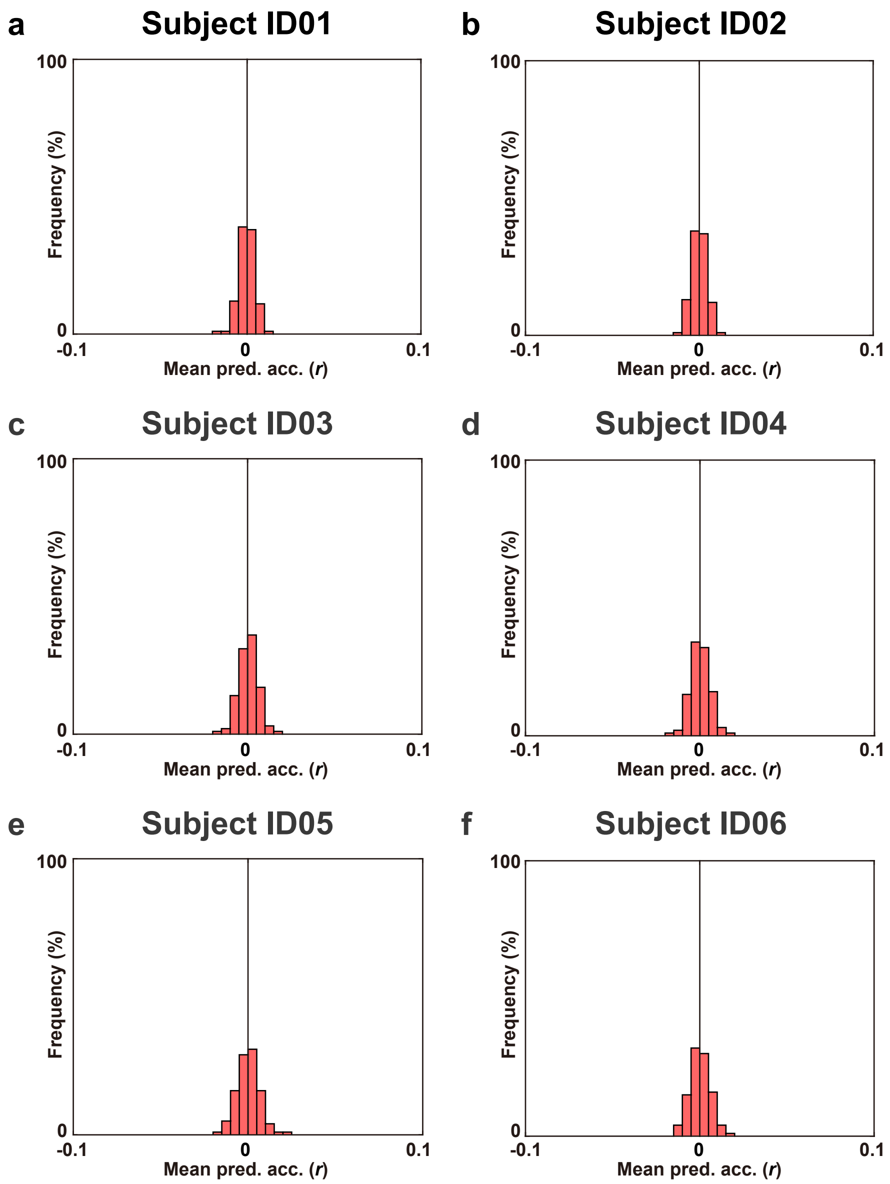

Supplementary Figure 12. Distribution of prediction accuracy with random shuffling. The histogram of mean prediction accuracy generated by a random shuffling procedure is plotted for subjects ID01–ID06.

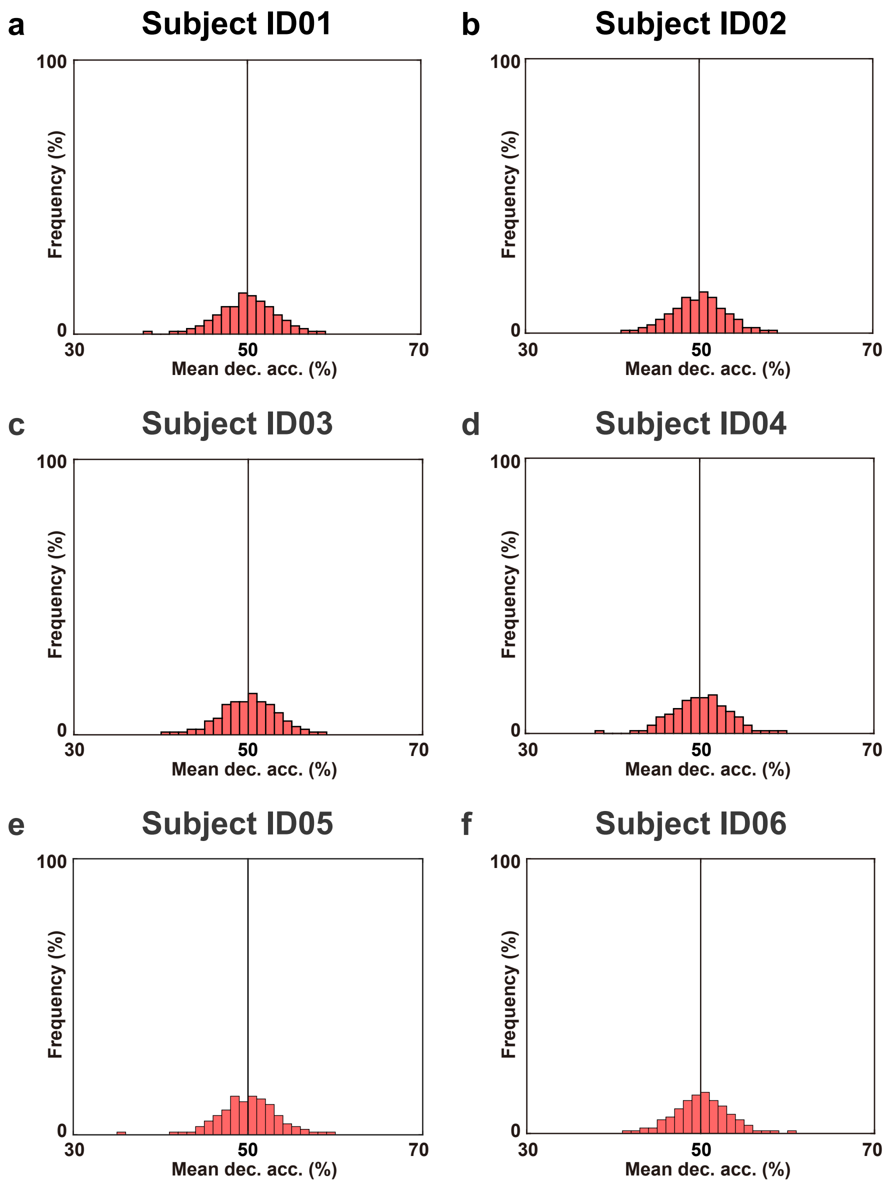

Supplementary Figure 13. Distribution of decoding accuracy with random shuffling. The histogram of mean decoding accuracy generated by a random shuffling procedure is plotted for subjects ID01–ID06.

Supplementary Note 8. Comparison between prediction and decoding results

The prediction accuracy was evaluated based on Pearson's correlation coefficient between predicted and actual responses (chance level = 0.0, Fig. 5b), whereas decoding accuracy was evaluated using one vs. one examinations based on the task likelihood (chance level = 50%, Fig. 6b). When we calculated the decoding accuracy using Pearson's correlation coefficient between the actual and decoded features (latent features of cognitive factor model), the decoding accuracy was 0.762 ± 0.019 (mean ± SD across six subjects).

The reported prediction accuracy (~0.3) was an average score of the whole cerebral cortex (~60,000 voxels, Fig. 5B). This includes regions that tend to have signal loss, such as the orbitofrontal cortex, and these regions may not contribute to the decoding performance.

To evaluate the contribution of informative voxels to the prediction and decoding performance, we selected the top 1000 voxels using the training dataset. For each subject, we randomly divided the training dataset into training samples (80%) and validation samples (20%) and performed model fitting using the cognitive factor model 50 times. The optimal regularization parameter was determined based on the mean prediction accuracy across the cortex and all repetitions. The top 1000 voxels that exhibited the largest prediction accuracy averaged across 50 repetitions were used in the following analyses. Note that the test dataset was independent of the voxel selection procedure.

When we used only the top 1000 voxels, the mean prediction accuracy of the test dataset (novel tasks) was 0.772 ± 0.039. The mean decoding accuracy was 95.8% ± 1.4% using task likelihood (0.720 ± 0.032 using Pearson's correlation coefficient). Therefore, prediction and decoding analyses produced a comparable performance.

Supplementary Methods

Description of each task

1. PressRight

Subjects pressed the buttons (with their right hand) as many times as possible. Duration: 8 s.

2. PressLeft

Subjects pressed the buttons (with their left hand) as many times as possible. Duration: 8 s.

3. PressLR

Subjects pressed the buttons (with their right or left hand) as many times as possible. Duration: 8 s.

4. RestOpen

Subjects did not perform any task and kept their eyes open. Duration: 10 s.

5. RestClose

Subjects did not perform any task and kept their eyes closed. Duration: 10 s.

6. EyeBlink

Subjects blinked their eyes as many times as possible. Duration: 8 s.

7. RateTired

Subjects rated how tired they were by pressing one of the four buttons. Duration: 6 s.

8. RateConfidence

Subjects rated how confident they were about their accuracy on the previous task by pressing one of the four buttons. Duration: 6 s.

9. RateSleepy

Subjects rated how sleepy they were by pressing one of the four buttons. Duration: 6 s.

10. ImagineFuture

Subjects imagined their future situation (e.g., “Imagine your next weekend”). Duration: 8 s.

11. ImagineIf

Subjects imagined they were some other living thing. Duration: 8 s.

12. ImagineMove

Subjects imagined their body moving. Duration: 8 s.

13. ImaginePlace

Subjects imagined a certain place. Duration: 8 s.

14. RecallPast

Subjects recalled a past event. Duration: 8 s.

15. RecallKnowledge

Subjects recalled as many names as possible that have a given property (e.g., recall as many Japanese river names as possible). Duration: 10 s.

16. RecallFace

Subjects recalled the face of somebody. Duration: 8 s.

17. LetterFluency

Subjects recalled as many words as possible starting with a given letter. Duration: 10 s.

18. CategoryFluency

Subjects recalled as many words as possible belonging to a given word category. Duration: 10 s.

19. Clock

Subjects looked at a photo of a clock and judged whether the indicated time matched the time displayed above the photo. Duration: 6 s.

20. AnimalPhoto

Subjects looked at a photo of an animal and judged whether its name matched the name displayed above the photo. Duration: 6 s.

21. AnimalVoice

Subjects listened to the voice of an animal and judged whether its name matched the name shown on the screen. Duration: 6 s.

22. Money
Subjects looked at a photo of money and judged whether the indicated amount matched the amount displayed above the photo. Duration: 8 s.

23. TrafficSign
Subjects looked at a photo of a traffic sign and judged whether its meaning matched the meaning indicated above the photo. Duration: 6 s.

24. EmotionFace
Subjects looked at a photo of a face with a specific emotion and judged whether the emotion matched the emotion indicated above the photo. Duration: 6 s.

25. EmotionVoice
Subjects listened to a voice with a specific emotion and judged whether the emotion matched the emotion indicated above the photo. Duration: 6 s.

26. Flag
Subjects looked at a photo of a national flag and judged whether the country matched the country indicated above the photo. Duration: 6 s.

27. MapSymbol
Subjects looked at a photo of a map symbol and judged whether its meaning matched the meaning indicated above the photo. Duration: 6 s.

28. CalcEasy
Subjects solved an easy arithmetic problem using single digits. Duration: 8 s.

29. CalcHard
Subjects solved a difficult arithmetic problem using two-digit numbers. Duration: 10 s.

30. DailyPhoto

Subjects looked at a photo of a tool used daily and judged whether its name matched the name displayed above the photo. Duration: 6 s.

31. DailySound

Subjects listened to the sound of tool used daily and judged whether its name matched the name displayed above the photo. Duration: 6 s.

32. CountDot

Subjects counted the number of presented dots. Duration: 8 s.

33. CountTone

Subjects counted the number of presented tones. Duration: 8 s.

34. CountryMap

Subjects looked at a photo of a country map and judged whether its name (nation) matched the name displayed above the photo. Duration: 6 s.

35. StateMap

Subjects looked at a photo of a state (prefecture) map and judged whether its name matched the name displayed above the photo. Duration: 6 s.

36. RateSexyPicF

Subjects looked at a photo of a female and rated how sexy they thought she was. Duration: 6 s.

37. RateSexyPicM

Subjects looked at a photo of a male and rated how sexy they thought he was. Duration: 6 s.

38. RateSexyMovM

Subjects viewed a movie of a male and rated how sexy they thought he was. Duration: 10 s.

39. RateSexyMovF

Subjects viewed a movie of a female and rated how sexy they thought she was. Duration: 10 s.

40. RateBeautyPic

Subjects looked at a photo and rated how beautiful they thought it was. Duration: 6 s.

41. RateBeautySound

Subjects listened to a piece of music and rated how beautiful they thought it was. Duration: 10 s.

42. RateBeautyMov

Subjects viewed a movie and rated how beautiful they thought it was. Duration: 10 s.

43. RateDisgustPic

Subjects looked at a photo and rated how disgusting they thought it was. Duration: 6 s.

44. RateDisgustSound

Subjects listened to a sound and rated how disgusting they thought it was. Duration: 6 s.

45. RateDisgustMov

Subjects viewed a movie and rated how disgusting they thought it was. Duration: 10 s.

46. RateHappyPic

Subjects looked at a photo and rated how happy the situation seemed to be. Duration: 6 s.

47. RateHappyMov

Subjects viewed a movie and rated how happy the situation seemed to be. Duration: 10 s.

48. RateDeliciousPic

Subjects saw a photo of food and rated how delicious it looked. Duration: 6 s.

49. RateDeliciousMov

Subjects viewed a movie of food and rated how delicious it looked. Duration: 10 s.

50. RatePainfulPic

Subjects looked at a photo and rated how painful the situation seemed to be. Duration: 6 s.

51. RatePainfulMov

Subjects viewed a movie and rated how painful the situation seemed to be. Duration: 10 s.

52. RateNoisy

Subjects listened to a sound and rated how noisy they thought it was. Duration: 8 s.

53. RatePoem

Subjects read a poem and rated how good they thought it was. Duration: 12 s.

54. WordMeaning

Subjects judged whether the meaning of a presented word matched the sentence displayed above the word. Duration: 6 s.

55. EyeMoveEasy

Subjects looked at a small circle moving around in 1 Hz. Duration: 8 s.

56. EyeMoveHard

Subjects looked at a small circle moving around at 2 Hz. Duration: 8 s.

57. WorldName

Subjects looked at the photo of a foreign celebrity and judged whether their name matched the name displayed above the photo. Duration: 6 s.

58. DomesticName

Subjects looked at the photo of a local celebrity and judged whether their name matched the name displayed above the photo. Duration: 6 s.

59. SoundPlace

Subjects listened to an environmental sound and judged whether it matched the location on the screen. Duration: 6 s.

60. WorldPlace

Subjects looked at a photo of a place in some foreign country and judged whether it matched the site displayed above the photo. Duration: 6 s.

61. DomesticPlace

Subjects looked at a photo of a place in their home country and judged whether it matched the site displayed above the photo. Duration: 6 s.

62. MusicCategory

Subjects judged whether the genre of a piece of music matched the name displayed on the screen. Duration: 10 s.

63. DetectTargetPic

Subjects judged whether a target item was shown in a photo. Duration: 8 s.

64. DetectTargetMov

Subjects judged whether a target item was shown in a movie clip. Duration: 10 s.

65. Metaphor

Subjects read a metaphorical text and judged whether the writer's intention matched the meaning indicated above the text. Duration: 8 s.

66. Sarcasm

Subjects read a sarcastic text and judged whether the writer's intention matched the meaning indicated above the text. Duration: 8 s.

67. TimeMov

Subjects judged whether the duration of a presented movie matched the duration indicated on the screen. Duration: 8 s.

68. TimeSound

Subjects judged whether the duration of a presented sound matched the duration indicated on the screen. Duration: 8 s.

69. ComparePeople

Subjects looked at two photos of people and judged whether or not the two were the same person. Duration: 6 s.

70. DetectDifference

Subjects looked at two pictures and judged whether or not they were exactly the same. Duration: 8 s.

71. Harmony

Subjects listened to a sequence of chords and judged whether the chord progression was consonant or dissonant. Duration: 6 s.

72. DecideFood

Subjects looked at four photos of different foods and judged which looked the most delicious. Duration: 8 s.

73. DecidePeople

Subjects looked at four photos of different people and judged who looked the most reliable. Duration: 8 s.

74. DecidePresent

Subjects chose one among four items they wanted to receive as a present. Duration: 8 s.

75. DecideShopping

Subjects chose one among four items they would buy during shopping. Duration: 8 s.

76. LanguageSound

Subjects listened to a sound and judged whether the language matched the language indicated on the screen. Duration: 6 s.

77. DetectColor

Subject judged whether the color of a word matched the color displayed above the word. Duration: 6 s.

78. SoundLeft

Subjects judged whether a sound was presented from their left side. Duration: 8 s.

79. SoundRight

Subjects judged whether a sound was presented from their right side. Duration: 8 s.

80. RelationLogic

Subjects read a syllogism based on spatial relationships and indicated whether the conclusion was valid or not. Duration: 12 s.

81. PropLogic

Subjects read a syllogism based on prepositional logical relationships and indicated whether the conclusion was valid or not. Duration: 12 s.

82. MoralPersonal

Subjects read a text and judged whether the described activity (which included harming somebody) was ethically permissible or not. Duration: 12 s.

83. MoralImpersonal

Subjects read a text and judged whether the described activity (which did not include harming somebody) was ethically permissible or not. Duration: 12 s.

84. Recipe

Subjects judged whether a given recipe matched the actual recipe of a given dish. Duration: 8 s.

85. TimeValue

Subjects selected one of two money rewards which would be offered to them at different points in the future. Duration: 8 s.

86. PressOrdEasy

Subjects pressed buttons based on a series of numbers presented at 1 Hz. Duration: 8 s.

87. PressOrdHard

Subjects pressed buttons based on a series of numbers presented at 2 Hz. Duration: 8 s.

88. Rhythm

Subjects listened to a series of sound pulses and judged whether its rhythm was constant or not. Duration: 6 s.

89. RecallTaskEasy
Subjects judged whether the two earlier tasks matched the task described on the screen. Duration: 6 s.

90. RecallTaskHard
Subjects judged whether the three earlier tasks matched the task described on the screen. Duration: 6 s.

91. MemoryDigit
Subjects memorized a series of digits. Duration: 6 s.

92. MatchDigit
Subjects judged whether a presented series of digits matched the one presented before (corresponding to the digits memorized in the MemoryDigit task). Duration: 6 s.

93. MemoryLetter
Subjects memorized a series of letters. Duration: 6 s.

94. MatchLetter
Subjects judged whether a presented series of letters matched the one presented before (corresponding to the letters memorized in the MemoryLetter task). Duration: 6 s.

95. MemoryNameEasy
Subjects memorized three names associated with three photos of different animal species. Duration: 6 s.

96. MatchNameEasy
Subjects judged whether two presented photos matched the names displayed on the screen (corresponding to the names memorized in the MemoryNameEasy task). Duration: 8 s.

97. MemoryNameHard
Subjects memorized three names associated with three photos of the same animal species. Duration: 6 s.

98. MatchNameHard

Subjects judged whether two presented photos matched the names displayed on the screen (corresponding to the names memorized in the MemoryNameHard task). Duration: 8 s.

99. ForeignRead

Subjects read an English sentence (i.e., a foreign language for the subjects). Duration: 12 s.

100. ForeignReadQ

Subjects answered a question about the English sentence they read just before. Duration: 6 s.

101. ForeignListen

Subjects listened to an English sentence. Duration: 10 s.

102. ForeignListenQ

Subjects answered a question about the English sentence they listened to just before. Duration: 6 s.

103. MirrorImage

Subjects judged whether a photo was symmetrical or not. Duration: 6 s.

Supplementary References

1. Yarkoni, T., Poldrack, R. A., Nichols, T. E., Van Essen, D. C. & Wager, T. D. Large-scale automated synthesis of human functional neuroimaging data. *Nat. Methods* **8**, 665–670 (2011).
2. Dale, A. M., Fischl, B. & Sereno, M. I. Cortical surface-based analysis: I. Segmentation and surface reconstruction. *Neuroimage* **9**, 179–194 (1999).
3. Fischl, B., Sereno, M. I. & Dale, A. M. Cortical Surface-Based Analysis II: inflation, flattening, and a surface-based coordinate system. *Neuroimage* **9**, 195–207 (1999).
4. Chang, C.-C. & Lin, C.-J. LIBSVM: A library for support vector machines. *ACM Trans. Intell. Syst. Technol.* **2**, 1–27 (2011).
