# Supplementary material for: Quantitative models reveal the organization of diverse cognitive functions in the brain
Source: Nat Commun. 2020 Mar 2;11:1142. doi: 10.1038/s41467-020-14913-w (PMC7052192; doi:10.1038/s41467-020-14913-w)
Supplement: Supplementary file 3 — Reporting Summary [file 41467_2020_14913_MOESM3_ESM.pdf]

nature research

Corresponding author(s): Shinji Nishimoto
Last updated by author(s): Nov 24, 2019

# Reporting Summary

Nature Research wishes to improve the reproducibility of the work that we publish. This form provides structure for consistency and transparency in reporting. For further information on Nature Research policies, see Authors & Referees and the Editorial Policy Checklist .

## Statistics

For all statistical analyses, confirm that the following items are present in the figure legend, table legend, main text, or Methods section.

| n/a | Confirmed | |
|---|---|---|
| ☐ | ☒ | The exact sample size (*n*) for each experimental group/condition, given as a discrete number and unit of measurement |
| ☐ | ☒ | A statement on whether measurements were taken from distinct samples or whether the same sample was measured repeatedly |
| ☐ | ☒ | The statistical test(s) used AND whether they are one- or two-sided<br>*Only common tests should be described solely by name; describe more complex techniques in the Methods section.* |
| ☒ | ☐ | A description of all covariates tested |
| ☐ | ☒ | A description of any assumptions or corrections, such as tests of normality and adjustment for multiple comparisons |
| ☐ | ☒ | A full description of the statistical parameters including central tendency (e.g. means) or other basic estimates (e.g. regression coefficient) AND variation (e.g. standard deviation) or associated estimates of uncertainty (e.g. confidence intervals) |
| ☐ | ☒ | For null hypothesis testing, the test statistic (e.g. *F*, *t*, *r*) with confidence intervals, effect sizes, degrees of freedom and *P* value noted<br>*Give P values as exact values whenever suitable.* |
| ☒ | ☐ | For Bayesian analysis, information on the choice of priors and Markov chain Monte Carlo settings |
| ☒ | ☐ | For hierarchical and complex designs, identification of the appropriate level for tests and full reporting of outcomes |
| ☐ | ☒ | Estimates of effect sizes (e.g. Cohen's *d*, Pearson's *r*), indicating how they were calculated |

*Our web collection on statistics for biologists contains articles on many of the points above.*

## Software and code

Policy information about availability of computer code

| | |
|---|---|
| Data collection | Siemens MAGNETOM TrioTim (Siemens, syngo MR B17) and Presentation (Neurobehavioral Systems, ver. 18.0) |
| Data analysis | We used MATLAB (MathWorks Inc., 2016b) to estimate model weights of a multivariate regression model (Nishimoto et al., 2011 Current Biology; Huth et al., 2012 Neuron). Visualization of the model weights on the cortical map was conducted using pycortex (https://github.com/gallantlab/pycortex). Hierarchical clustering analysis and principal component analysis were performed using the standard MATLAB functions (dendrogram and pca). |

For manuscripts utilizing custom algorithms or software that are central to the research but not yet described in published literature, software must be made available to editors/reviewers. We strongly encourage code deposition in a community repository (e.g. GitHub). See the Nature Research guidelines for submitting code & software for further information.

## Data

Policy information about availability of data

All manuscripts must include a data availability statement. This statement should provide the following information, where applicable:
- Accession codes, unique identifiers, or web links for publicly available datasets
- A list of figures that have associated raw data
- A description of any restrictions on data availability

The data that support the findings of this study are available at OpenNeuro.org (raw MRI data; https://openneuro.org/datasets/ds002306) and Open Science Framework (preprocessed, figure source data, and codes; https://osf.io/ea2jc/)

# Field-specific reporting

Please select the one below that is the best fit for your research. If you are not sure, read the appropriate sections before making your selection.

☐ Life sciences ☒ Behavioural & social sciences ☐ Ecological, evolutionary & environmental sciences

For a reference copy of the document with all sections, see nature.com/documents/nr-reporting-summary-flat.pdf

# Behavioural & social sciences study design

All studies must disclose on these points even when the disclosure is negative.

| | |
|---|---|
| Study description | quantitative experimental |
| Research sample | Research participants were Osaka University students and adjuncts (4 males and 2 females, aged 22-33). All participants were healthy, had normal vision and hearing ability, and passed institutional pre-screening procedure for MRI experiments (e.g., no metal implants, not pregnant, etc.) |
| Sampling strategy | The participants were selected based on convenience (who can participate MRI sessions over multiple days) and gender balance. Data analysis and statistics were examined and confirmed for each participant separately by recording independent training and test datasets. The sample size of the test data (412 samples or 103 tasks) was determined to perform proper predictive and statistical analysis for encoding and decoding models and to match our prior attempts (e.g., Nishimoto et al., 2011 Current Biology) |
| Data collection | Data were collected using MRI scanner, button-box, video projector, and audio equipment. MRI operators were involved in MRI data collection. Researchers designed experiments and were not blind to experimental condition. |
| Timing | Aug. 12, 2017 - Jan. 5, 2018 |
| Data exclusions | Some data were excluded (and re-measured) when we detected the following technical issues during experiments: the earphone was not properly attached. |
| Non-participation | No participants dropped out. |
| Randomization | n/a (Participants were not allocated into experimental groups.) |

# Reporting for specific materials, systems and methods

We require information from authors about some types of materials, experimental systems and methods used in many studies. Here, indicate whether each material, system or method listed is relevant to your study. If you are not sure if a list item applies to your research, read the appropriate section before selecting a response.

## Materials & experimental systems

| n/a | Involved in the study | |
|---|---|---|
| ☒ | ☐ | Antibodies |
| ☒ | ☐ | Eukaryotic cell lines |
| ☒ | ☐ | Palaeontology |
| ☒ | ☐ | Animals and other organisms |
| ☐ | ☒ | Human research participants |
| ☒ | ☐ | Clinical data |

## Methods

| n/a | Involved in the study | |
|---|---|---|
| ☒ | ☐ | ChIP-seq |
| ☒ | ☐ | Flow cytometry |
| ☐ | ☒ | MRI-based neuroimaging |

## Human research participants

Policy information about studies involving human research participants

| | |
|---|---|
| Population characteristics | (See above) |
| Recruitment | Participants were recruited from a local participant pool under the following selection criteria: (1) a participant can join at least three fMRI sessions and (2) a participant is healthy and with normal vision and hearing. |
| Ethics oversight | National Institute of Information and Communications Technology |

Note that full information on the approval of the study protocol must also be provided in the manuscript.

# Magnetic resonance imaging

## Experimental design

| | |
|---|---|
| Design type | Building voxel-wise encoding models using task-evoked brain activity (Nishimoto et al., 2011 Current Biology) |
| Design specifications | The main experiment was conducted in three separate fMRI sessions. The total of 18 runs were acquired across the three sessions. Of these, 12 runs were used to train voxel-wise models, and 6 runs were used to test the modeling accuracy. A single run consisted of 556 seconds. Stimuli used in the training and test runs were different. |
| Behavioral performance measures | For 48 out of 103 tasks, task performance was measured using button responses and examined for each participant separately by their median and interquartile range. |

## Acquisition

| | |
|---|---|
| Imaging type(s) | functional and structural |
| Field strength | 3T |
| Sequence & imaging parameters | Functional data: A multiband gradient echo-planar imaging sequence (TR = 2,000 ms, TE = 30 ms, flip angle = 62°; voxel size = 2 × 2 × 2 mm3, matrix size = 96 × 96, 72 axial slices, FOV = 192 × 192 mm2, multiband factor = 3).<br>Structural data: T1-weighted MPRAGE (TR = 2530 ms, TE = 3.26 ms, flip angle = 9°, voxel size = 1 × 1 × 1 mm3, matrix size = 256 × 256, 256 axial slices, FOV = 256 × 256 mm2). |
| Area of acquisition | A whole brain scan was used |

Diffusion MRI ☐ Used ☒ Not used

## Preprocessing

| | |
|---|---|
| Preprocessing software | SPM8 (motion correction) and FreeSurfer 5.3.0 (anatomical registration, cortical surface reconstruction, cortical segmentation, and subcortical segmentation) |
| Normalization | n/a (data for each participant were analyzed individually) |
| Normalization template | n/a (data for each participant were analyzed individually) |
| Noise and artifact removal | Motion correction (6DOF) was performed by aligning all of the EPI data to the first image from the first scan for each subject. For each voxel, responses were normalized by subtracting the mean response across all time points, and trend was removed using a median filter (240-s time window). These processes were performed using in-house MATLAB codes (Cukur et al., 2016 The Journal of Neuroscience). No spatial smoothing procedure was performed. |
| Volume censoring | n/a (no censoring was performed and all data were used for the study) |

## Statistical modeling & inference

| | |
|---|---|
| Model type and settings | Mass univariate, predictive, and RSA. Feature-based encoding models were built using the training data sets, and the modeling accuracy was examined by using the separate test datasets. |
| Effect(s) tested | Prediction accuracy (measured by correlation coefficients) and decoding accuracy (measured by the number of accurate choice) under novel task conditions |

Specify type of analysis: ☒ Whole brain ☐ ROI-based ☐ Both

| | |
|---|---|
| Statistic type for inference (See Eklund et al. 2016) | voxel-wise |
| Correction | False-discovery rate (FDR) correction (Benjamini and Hochberg, 1995). |

## Models & analysis

| n/a | Involved in the study |
|---|---|
| ☒ | ☐ Functional and/or effective connectivity |
| ☒ | ☐ Graph analysis |
| ☐ | ☒ Multivariate modeling or predictive analysis |

| | |
|---|---|
| Multivariate modeling and predictive analysis | We built voxel-wise encoding models (Naselaris et al., 2011 NeuroImage; Nishimoto et al., 2011 Current Biology) to explain the BOLD responses using task-related features. Model weights were estimated using a L2-regularized linear regression procedure (Huth et al., 2012 Neuron) for training data (3336 samples). The regularization parameter was optimized via 10-fold cross validation using the training data. The prediction |

accuracy of each voxel model was quantified by a Pearson's correlation coefficients between the measured and the predicted BOLD responses for test data (412 samples).
